# Acylated- and unacylated ghrelin during an oral glucose tolerance test in humans at risk for type 2 diabetes mellitus

**DOI:** 10.1038/s41366-023-01327-z

**Published:** 2023-07-07

**Authors:** Magnus Wolf, Martin Heni, Anita M. Hennige, Katrin Sippel, Alexander Cegan, Lina María Serna Higuita, Peter Martus, Hans-Ulrich Häring, Andreas Fritsche, Andreas Peter

**Affiliations:** 1 Department for Diagnostic Laboratory Medicine, Institute for Clinical Chemistry and Pathobiochemistry, University Hospital of Tübingen, Tübingen, Germany; 2 Institute for Diabetes Research and Metabolic Diseases (IDM) of the Helmholtz Center Munich at the University of Tübingen, Tübingen, Germany; 3 German Center for Diabetes Research (DZD), München-Neuherberg, Germany; 4 Department for Diagnostic Laboratory Medicine, Institute for Medical Virology and Epidemiology of Viral Diseases, University Hospital of Tübingen, Tübingen, Germany; 5 Department for Internal Medicine IV, Division for Diabetology, Endocrinology and Nephrology, University Hospital of Tübingen, Tübingen, Germany; 6 Division of Endocrinology and Diabetology, Department of Internal Medicine 1, University Hospital Ulm, Ulm, Germany; 7 Boehringer Ingelheim International GmbH, Biberach, Germany; 8 Department of Biological and Biochemical Sciences, Faculty of Chemical Technology, University of Pardubice, Pardubice, Czech Republic; 9 Institute for Clinical Epidemiology and applied Biostatistics, University of Tübingen, Tübingen, Germany

**Keywords:** Obesity, Pre-diabetes, Gastrointestinal hormones

## Abstract

**Background/Objectives:**

The orexigenic peptide hormone ghrelin has been implicated in the pathophysiology of obesity and type 2 diabetes mellitus through its effects on nutrient homeostasis. Ghrelin is subject to a unique post-translational acyl modification regulating its biochemical activity.

**Subjects/Methods:**

In this study we aimed to investigate the relation of acylated (AcG) as well as unacylated ghrelin (UnG) with body weight and insulin resistance in the fasting (*n* = 545) and post-oral glucose tolerance test (oGTT) state (*n* = 245) in a metabolically well characterized cohort covering a broad range of BMI (17.95 kg/m²–76.25 kg/m²).

**Results:**

Fasting AcG (median 94.2 pg/ml) and UnG (median 175.3 pg/ml) were negatively and the AcG/UnG ratio was positively correlated with BMI (all *p* < 0.0001). Insulin sensitivity (ISI) correlated positively with AcG (*p* = 0.0014) and UnG (*p* = 0.0004) but not with the AcG/UnG ratio. In a multivariate analysis, including ISI and BMI, only BMI, but not ISI was independently associated with AcG and UnG concentrations. Significant changes of AcG and UnG concentrations were detectable after oGTT stimulation, with slight decreases after 30 min and increases after 90–120 min. Subject stratification into BMI-divergent groups revealed more pronounced AcG increases in the two groups with BMI < 40 kg/m².

**Conclusion:**

Our data demonstrate lower concentrations for both AcG and UnG with increasing BMI as well as an increased proportion of the biologically active, acylated form of ghrelin giving point to pharmacologic intervention in ghrelin acylation and/or increase in UnG for treatment of obesity despite decreased absolute AcG levels.

## Introduction

Obesity is caused by a dysregulation of energy balance due to multiple factors including gastrointestinal hormones. It leads to a variety of complications such as type 2 diabetes mellitus (T2DM) and cardiovascular disease, and the prevalence is rising worldwide. The orexigenic gastric peptide hormone ghrelin is a regulator of appetite and plays a role in controlling energy intake [1–3]. Alterations are thought to be involved in the pathogenesis of obesity and related diseases [4–6]. Furthermore, ghrelin is implicated in the promotion of glucose-stimulated insulin secretion [7] as well as the regulation of insulin sensitivity [8] an substrate metabolism in adipose- and skeletal muscle tissues [9]. Hence, ghrelin appears to also affect carbohydrate metabolism directly, in addition to its indirect effects *via* promotion of food intake, obesity and subsequent insulin resistance. Thus, ghrelin could be a promising drug target for multiple aspects of metabolic diseases.

Ghrelin is primarily produced by the proximal enteroendocrine X/A-like cells in the gastric fundus submucosa [4, 10, 11] and in small amounts in locations such as the hypothalamus [3]. Ghrelin action is primarily detectable in the arcuate nucleus in the brain [4]. The regulation of ghrelin action includes multiple post-translational modifications like the cleavage of proghrelin to 28-amino acid ghrelin and the octanoylation of ghrelin (UnG) at serine residue 3 [11–13] by the enzyme ghrelin *O*-acyl transferase (GOAT) [14] yielding acylated ghrelin (AcG). This unique modification for peptide hormones is required for ghrelin’s ability to activate its receptor, the growth hormone secretagogue receptor (GHSR) 1a but also impairs the receptor independent transport across the blood-brain barrier [15]. Inhibition of ghrelin acylation by intraperitoneal administration of a GOAT inhibitor improves glucose tolerance in mice [16] and UnG has been shown to improve muscle oxidative metabolism and performance [17] suggesting GHSR independent effects of UnG. Furthermore, overexpression of UnG results in improved vascular function [18].

Circulating levels of ghrelin increase in the fasting state and are postprandially suppressed by food intake with strongest effects of proteins and carbohydrates [5, 19, 20]. Intracerebroventricular administration of the orexigenic hormone ghrelin increases food ingestion and body weight in rodents [3, 5]. Consistent with a negative feedback regulation in response to positive energy balance, ghrelin levels are decreased in obesity [5] and under conditions of insulin resistance [6] in humans. In contrast, increased circulating ghrelin levels are found in patients with restricting type of anorexia nervosa [21, 22]. Furthermore, ghrelin action has recently been linked to addiction, including alcohol addiction [23–25], binge eating disorders [26], cessation of smoking [27] as well as persistent gambling [28]).

Unfortunately, most available studies only report total ghrelin levels and do not differentiate between the biochemically active acylated (AcG) and unacylated ghrelin (UnG).

Kuppens reported increases in the AcG / UnG ratio to be associated with the onset of hyperphagia in individuals with Prader-Willi syndrome [29] and in a small study of adults with Obesity, Barazzoni suggested that obesity could influence the AcG / UnG ratio and a relative AcG -excess could contribute to obesity-associated insulin resistance [30].

In the present study, we therefore analyzed both, AcG and UnG, in a large cohort of metabolically well-characterized individuals covering a large BMI range at baseline as well as after oral glucose ingestion to address the questions if, first, AcG is also diminished in obesity and whether the proportion of AcG and UnG is altered, second circulating ghrelin (AcG and UnG) is associated with insulin resistance independent of obesity and third, post-glucose (oGTT) ghrelin alterations are associated with obesity or insulin resistance.

Finally, since the gene product of the fat mass- and obesity-associated gene (*FTO*) was proposed as regulator for ghrelin levels and ghrelin action [1, 31], we aimed to elucidate effects of the *FTO* SNP rs8050136 on ghrelin concentrations.

## Research design and methods

### Study cohort

The study cohort of this present work consisted of 545 Caucasians from the southern part of Germany who had participated in studies on the pathophysiology and prevention of T2DM conducted at the University hospital of Tübingen [32] fulfilling at least one of the following criteria: family history of T2DM, body mass index (BMI) > 27 kg/m^2^, previous diagnosis of impaired glucose tolerance or gestational diabetes. They were considered healthy according to a physical examination and routine laboratory tests. Stabilized blood samples collected at five time points before and during an oGTT were available for 245 of these subjects and were used for the analysis of ghrelin kinetics during the oGTT.

Informed written consent was obtained from all participants and the Ethics Committee of the University of Tübingen had approved the protocol.

### Oral glucose tolerance test

Venous plasma samples were obtained at five time points before and during a 75 g oGTT (0 min, 30 min, 60 min, 90 min and 120 min) for determination of plasma glucose and insulin concentrations. Insulin sensitivity index (ISI) was calculated from the oGTT measurements as proposed by Matsuda and DeFronzo [33].

### Analytical procedures

Blood glucose was determined using a bedside glucose analyser (glucose oxidase method; YSI, Yellow Springs Instruments, Yellow Springs, CO, USA). For all other measurements, blood was placed on ice immediately after drawing and transferred to the laboratory for subsequent centrifugation and analysis or storage at −80 °C until further analysis.

Plasma insulin was determined on the ADVIA Centaur® XPT chemiluminometric immunoassay system (Siemens Healthineers, Eschborn, Germany) and glycated hemoglobin (HbA1c) was determined using the Tosoh G8 HPLC analyzer (Tosoh Bioscience, Griesheim, Germany).

Measurements of AcG levels as well as UnG levels were performed with acidified and PMSF-stabilized EDTA plasma samples without refreezing the aliquots using Enzyme Immunoassay (EIA) kits from Bertin Pharma (Bertin Technologies SAS, Montigny-le-Bretonneux, France). All measurements were performed applying the same batch of reagents with a predilution of 1:5 for AcG and 1:10 for UnG (as recommended by the manufacturer) using the fully automated microtitration plate (MTP) analyzer system BEP^®^ 2000 Advance (Siemens Healthineers, Eschborn, Germany), adapted to the needs of the tests under supervision of the assay manufacturer. Measurements were performed in technical duplicates from the same pre-dilution.

### Single nucleotide polymorphism (SNP) genotyping

DNA was isolated from whole blood samples for genotyping using a commercial DNA isolation kit (NucleoSpin^®^, MACHEREY-NAGEL GmbH & Co. KG, Düren, Germany). The fat mass- and obesity-associated (*FTO*) gene polymorphism rs8050136 was genotyped in 521 subjects using the MassARRAY^®^ System by Agena Bioscience^TM^ with the iPLEX^®^ software (Agena Bioscience GmbH, Hamburg, Germany) as described elsewhere [34].

### Statistical analyses

Parameters whose distribution showed a large skewness resembling a log-normal distribution were logarithmically transformed for statistical analyses and are expressed as geometric mean ± geometric standard deviation (SD), whereas parameters whose distribution resembled neither normal nor log-normal distribution are expressed as median (25th percentile; 75th percentile). Transformation of baseline data for correlation and multivariate regression analyses was selected based on the best fitting with regard to the skewness and the kurtosis of the corresponding distribution. The skewness and the kurtosis of all distributions of the transformed baseline data, mentioned in this publication, were in-between −0.5 and +0.3 and in-between −0.6 and +0.6, respectively.

Univariate associations between parameters were tested using Pearson correlation analyses. Multivariate linear regression analyses were applied both to adjust for potential covariates and to identify independent relationships. The statistical significance of differences between the three BMI groups in the levels of AcG, UnG or the AcG/UnG ratio as well as in their AUCs during oGTT was analyzed by nonparametric unpaired two-tailed Wilcoxon tests with an alpha level of 0.05.

Kinetics of the total cohort during oGTT were investigated by a pre-hoc one-way repeated measures analysis of variance (ANOVA) by ranks (Friedman tests). Subsequently, the significance of differences between the fasting state (0 min) and the other four oGTT time points was assessed by post-hoc nonparametric paired two-tailed Dunn’s tests and with the alternative hypothesis that at least one of the four oGTT time points (30 min, 60 min, 90 min, 120 min) differs from the corresponding fasting state (0 min). Genotype effects of the *FTO* gene polymorphism rs8050136 on ghrelin concentrations were analyzed using an additive model.

The statistical software package JMP^®^ version 13.0.0 (SAS Institute Inc., Cary, NC, USA) was applied for Shapiro-Wilk W tests, univariate association studies, multivariate linear regression analyses and nonparametric unpaired Wilcoxon tests. Pre-hoc Friedman tests as well as post-hoc paired Dunn’s tests for investigation of ghrelin kinetics during oGTT were performed using the alternative statistical software package Prism^®^ version 7.03 (GraphPad Software, Inc., La Jolla, CA, USA). *P*-values < 0.05 were considered statistically significant.

## Results

Subject characteristics and anthropometric data of the total cohort of 545 individuals are presented in Table 1.

**Table 1:** Anthropometrics, parameters of glucose homeostasis, levels of acylated ghrelin (AcG), unacylated ghrelin (UnG) and their ratio (AcG/UnG) in the total cohort with results from 545 subjects.

PARAMETER	TOTAL COHORT (*n* = 545)
	Median (25th perc.; 75th perc.) or geometric mean ± geometric SD
**BMI** [kg/m^2^]	34.1 (27.9; 42.4)
**Age** [years]	49.0 (39.0; 60.0)
**Gender** (♂/♀)	208/337
**AcG (fasting)** [pg/ml]	94.2 (54.0; 154.9)
**UnG (fasting)** [pg/ml]	175.3 (97.3; 337.4)
**AcG/UnG (fasting)** [-]	0.48 (0.34; 0.75)
**Glucose (fasting)** [mmol/l]	5.33 (5.00; 5.72)
**HbA1c** [%]	5.7 (5.4; 5.9)
**ISI-Matsuda** [AU]	8.02 ± 1.95

Results are presented as geometric mean ± geometric standard deviation (SD) in case of log-normally- distributed data or as median (25th percentile; 75th percentile) for nonnormally distributed data.

The detected fasting concentrations of AcG (94.2 pg/ml) and UnG (175.3 pg/ml) in this cohort were comparable to those reported previously in healthy subjects [19]. No gender differences of AcG (*p* = 0.6155) or UnG (*p* = 0.2060) concentrations, as well as the AcG/UnG ratio (*p* = 0.3598) were detected in accordance with previous reports [14].

**Fig. 1:**
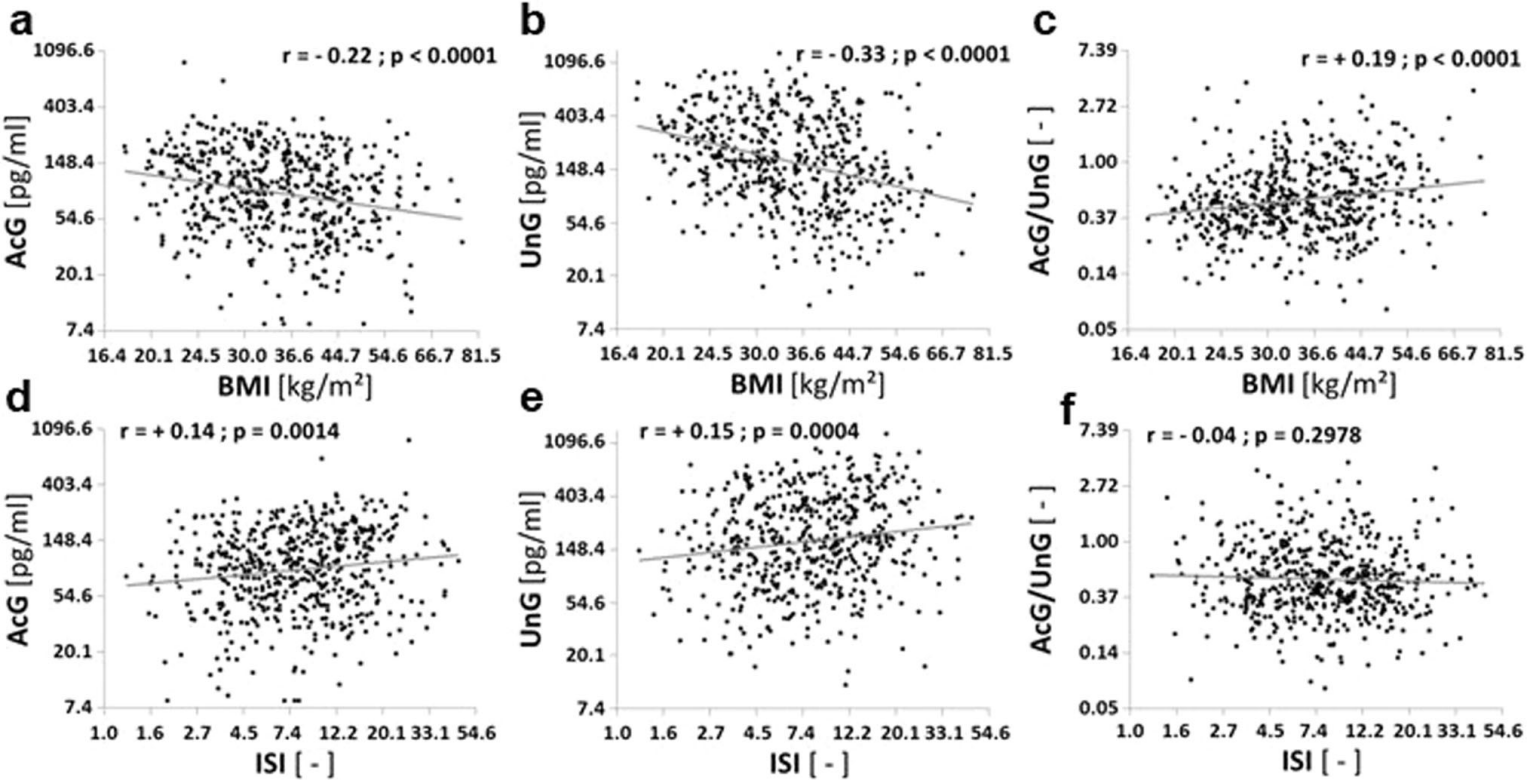
Ghrelin concentrations correlate with BMI and insulin sensitivity. Correlation analyses for fasting concentrations of acylated ghrelin (AcG; **a**, **d**), unacylated ghrelin (UnG; **b**, **e**) and their ratio (AcG/UnG; **c**, **f**) in the total cohort (*N* = 545) with body mass index (BMI; **a**–**c**) or Matsuda’s insulin sensitivity index (ISI; **d**–**f**).

Next, we performed correlation analyses of ghrelin concentrations with BMI (Fig. 1a–c) and ISI (Fig. 1d–f) revealing negative correlations for AcG levels (*p* < 0.0001, *r* = −0.22) as well as for UnG levels (*p* < 0.0001, *r* = −0.33) with BMI, whereas the AcG/UnG ratio was positively correlated with BMI (*p* < 0.0001, *r* = +0.19). Matsuda’s ISI correlated positively with AcG levels (*p* = 0.0014, *r* = +0.14) and UnG levels (*p* = 0.0004, *r* = +0.15) but not with the AcG/UnG ratio (*p* = 0.30). The correlations of BMI with ghrelin concentrations AcG, UnG as well as the AcG/UnG ratio were independent of ISI in a multivariate linear regression model including both, ISI and BMI (Table 2). However, the strong correlations of both, AcG and UnG with ISI were abolished after adjustment for BMI, whereas the association of the AcG/UnG ratio with ISI was marginally significant (*p* = 0.033).

**Table 2:** Multivariate analyses for evaluation of potential associations of fasting levels of acylated ghrelin, unacylated ghrelin or their ratio with Matsuda’s insulin sensitivity index (ISI) and BMI in univariate and multivariate models.

	Association with insulin sensitivity index (ISI)		Association with BMI	
	univariate	BMI-adjusted	univariate	ISI-adjusted
**acylated ghrelin**	*p* = 0.0014; ß_std_ = 0.1366	*p* = 0.96; ß_std_ = 0.0026	*p* < 0.0001; ß_std_ = −0.2215	*p* < 0.0001; ß_std_ = −0.2199
**unacylated ghrelin**	*p* = 0.0004; ß_std_ = 0.1516	*p* = 0.11; ß_std_ = −0.0809	*p* < 0.0001; ß_std_ = −0.3322	*p* < 0.0001; ß_std_ = −0.3815
**ratio AcG/UnG**	*p* = 0.30; ß_std_ = −0.0447	*p* = 0.033; ß_std_ = 0.1130	*p* < 0.0001; ß_std_ = 0.1899	*p* < 0.0001; ß_std_ = 0.2587

We next analyzed the alteration of circulating ghrelin in response to an oral glucose load at five time points during an oGTT in a subgroup of 245 subjects with available suitably stabilized samples. Subject characteristics and anthropometric data of this oGTT subcohort are presented in the first columns of Table 3.

**Table 3:** Anthropometrics, ghrelin levels as acylated ghrelin (AcG), unacylated ghrelin (UnG) and their ratio (AcG/UnG) as well as parameters of glucose homeostasis in the subcohort of subjects from whom repetitive samples were available during an oral glucose tolerance test (oGTT).

	oGTT subcohort (*n* = 245)	normal/overweight BMI (*n* = 98)	high BMI (*n* = 83)	very high BMI (*n* = 64)	ANOVA
**BMI** [kg/m^2^]	32.1 (26.1; 40.4)	25.1 (22.8; 27.3)	33.6 (31.8; 36.4)	44.0 (42.4; 48.2)	<0.0001
**Age** [years]	56.0 (44.5; 63.5)	62.0 (56.8; 66.3)	56.0 (46.0; 63.0)	44.5 (36.3; 49.8)	<0.0001
**Gender** (♂/♀)	84/161	40/58	29/54	15/49	
**AcG (fasting)** [pg/ml]	115.8 (70.7; 175.5)	136.3 (78.5; 180.6)	122.7 (71.4; 176.2)	96.0 (54.9; 142.1)	0.0117
**UnG (fasting)** [pg/ml]	285.6 (170.8; 458.5)	327.5 (190.8; 494.2)	286.6 (168.2; 441.4)	213.1 (128.4; 373.8)	0.0110
**AcG/UnG (fast.)** [-]	0.38 (0.30; 0.53)	0.37 (0.29; 0.50)	0.40 (0.31; 0.51)	0.38 (0.30; 0.56)	0.2267
**Glucose (fast.)** [mmol/l]	5.33 (5.06; 5.61)	5.17 (5.00; 5.44)	5.34 (5.06; 5.78)	5.44 (5.02; 5.67)	0.0330
**HbA1c** [%]	5.8 (5.5; 6.0)	5.8 (5.6; 6.0)	5.7 (5.5; 6.0)	5.7 (5.4; 5.9)	0.2420
**ISI-Matsuda** [AU]	7.52 ± 1.81	10.89 ± 1.57	6.94 ± 1.64	4.74 ± 1.74	<0.0001

Characteristics are also presented for three BMI-stratified subgroups. Ghrelin levels are specified in. Results are presented as geometric mean ± geometric standard deviation (SD) in case of log-normally- distributed data or as median (25th percentile; 75th percentile) for nonnormally distributed data.

**Fig. 2:**
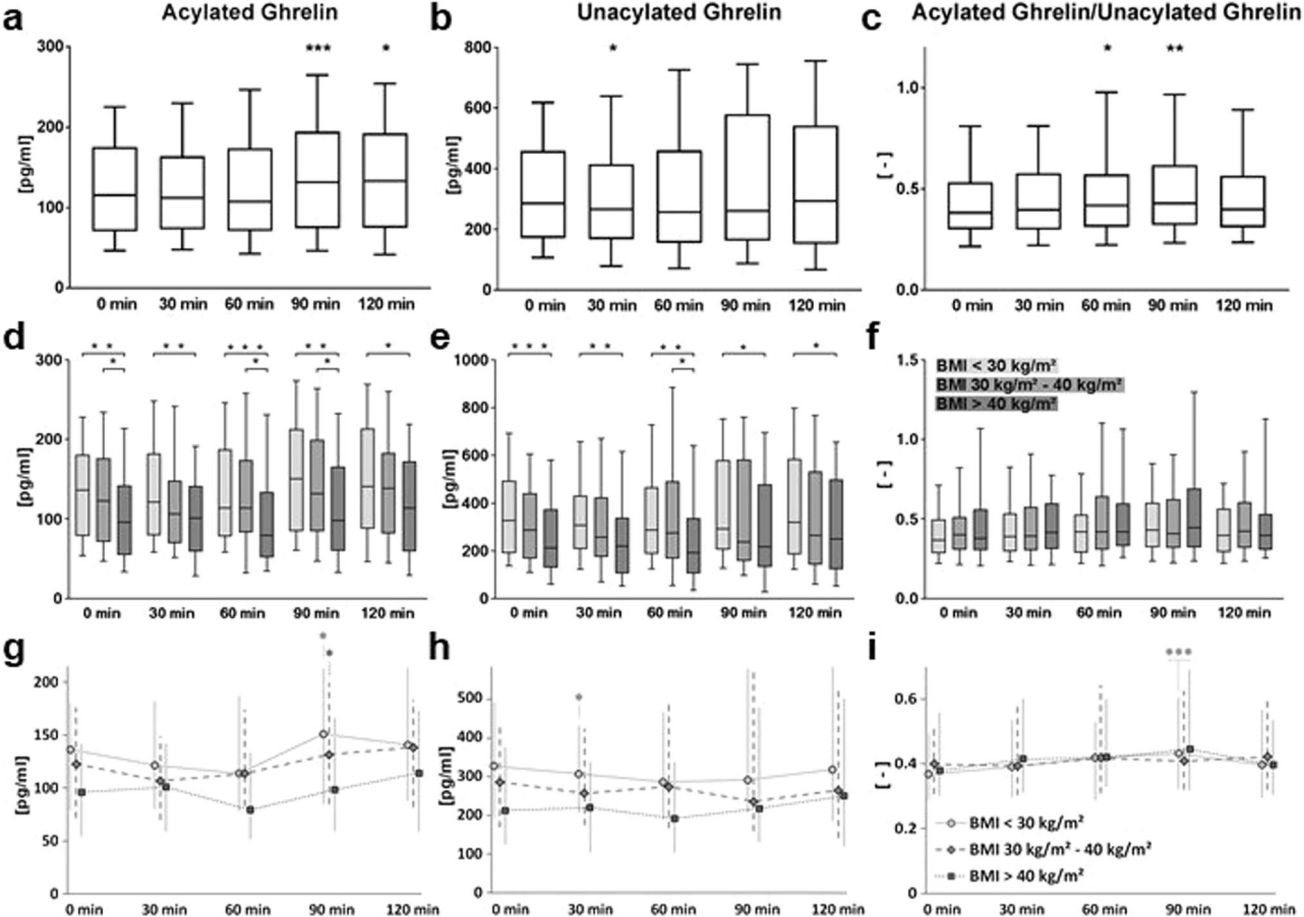
Changes of ghrelin concentrations after an oral glucose challenge. Kinetics of the concentrations of acylated ghrelin (**a**, **d**, **g**), unacylated ghrelin (**b**, **e**, **h**) and their ratio (**c**, **f**, **i**) before (0 min) and during (30 min, 60 min, 90 min, and 120 min) an oral glucose tolerance test (oGTT) in the oGTT subcohort (*N* = 245) without stratification (**a**–**c**) and with body mass index (BMI)-dependent stratification of the subjects into a normal/overweight BMI (pale gray; BMI < 30 kg/m²; *N* = 98), a high BMI (medium gray; BMI 30 kg/m²–40 kg/m²; *N* = 83) and a very high BMI (deep gray; BMI > 40 kg/m²; *N* = 64) group (**d**–**i**). Significance of d**i**fferences between the three BMI groups was tested by unpaired nonparametric Wilcoxon tests (**d**–**f**), whereas the signi**f**icance of differences of the four oGTT time points (30 min, 60 min, 90 min and 120 min) in comparison to baseline (0 min) in total oGTT subcohort (**a**–**c**) as well as in e**a**ch of the three BMI-stratified groups (**g**–**i**) was evaluated by pa**i**red nonparametric Friedman’s tests. The boxes of the graphs (**a**–**f**) represent the median value plus the 25th percentile and the 75th percentile, the whiskers visualize the 10th percentile and the 90th percentile, respectively. The lines of the graphs (**g**–**i**) connect the median values, the error bars show the lower quartile and the upper quartile, respectively. *p ≤ 0.05; **p ≤ 0.01; ***p ≤ 0.001.

Nonparametric one-way ANOVA with repeated measures design by ranks (Friedman tests) revealed highly significant changes during the oGTT (AcG: *p* < 0.0001; UnG: *p* = 0.0007; ratio: *p* = 0.0087), but as specified by post-hoc Dunn’s Tests, in contrast to our expectations, we did not observe a strong decrease in circulating ghrelin levels after glucose ingestion. As depicted in Fig. 2a, b, we rather detected an unanticipated significant increase in median AcG after 90 min (*p* = 0.0009, +13.6%) and 120 min (*p* = 0.0158, +14.9%) over fasting concentrations (Fig. 2a). Only UnG (Fig. 2b) significantly decreased at early time points of the oGTT (30 min: *p* = 0.0321, −6.8 %) but also tended to increase at later time points (120 min: *p* = 0.0719, +2.6%). The AcG/UnG ratio (Fig. 2c) significantly increased after 60 min and 90 min (*p* = 0.0164, +9.4% and *p* = 0.0086, +12.1%, respectively). Notably, the individual ghrelin concentrations at all time points as well as the response to the glucose ingestion exhibit broad interindividual variations that were also observed by other groups [14].

To investigate the effect of body weight on ghrelin kinetics during the oGTT, the subcohort was stratified into three BMI-diverging groups (normal/overweight BMI < 30 kg/m² or high BMI 30 kg/m² −40 kg/m² or very high BMI > 40 kg/m²) which are characterized in the last columns of Table 3. The ghrelin kinetics in these three BMI-diverging groups during oGTT are illustrated in Fig. 2d–i.

Comparisons of the ghrelin levels between the three BMI-diverging groups by nonparametric unpaired Wilcoxon tests at the five different time points during oGTT revealed significant decreases of both AcG levels (Fig. 2d) and UnG levels (Fig. 2e) at all five time points before and during oGTT for the group with BMI > 40 kg/m² in comparison to the group with BMI < 30 kg/m² and mostly also in comparison to the group with BMI in between 30 kg/m² and 40 kg/m². The AcG/UnG ratio, however, was not significantly different between the BMI groups (*p* > 0.3; Fig. 2f).

BMI-group-separated analysis of nonparametric one-way ANOVAs with repeated measures design by ranks (Friedman tests) showed significant kinetic changes during oGTT for the group with BMI < 30 kg/m² (AcG: *p* = 0.0042; UnG: *p* = 0.0396; ratio: *p* = 0.0055) as well as for the group with BMI in between 30 kg/m² and 40 kg/m² (AcG: *p* = 0.0367; UnG: *p* = 0.0151; ratio: *p* = 0.1139) but not for the group with BMI > 40 kg/m² (AcG: *p* = 0.1578; UnG: *p* = 0.3223; ratio: *p* = 0.7289).

Statistically significant pre-hoc tests were specified by post-hoc paired Dunn’s tests for each oGTT time point compared to baseline without correction (Fig. 2g, h): The group with BMI < 30 kg/m² showed an AcG increase after 90 min (*p* = 0.0147; median increase: +10.7%), an UnG decline after 30 min (*p* = 0.0421; median decrease: −6.1%) and an increase of the ratio after 90 min (*p* = 0.0005; median increase: +17.3%), whereas the group with BMI in between 30 kg/m² and 40 kg/m² showed solely a significant AcG increase after 90 min (*p* = 0.0255; median increase: +7.2%) but no significant differences from baseline for UnG during oGTT (*p* ≥ 0.0858) in post-hoc testing.

Thus, the BMI-group-specific evaluation revealed significant dynamic changes of circulating ghrelin kinetics during the oGTT that were blunted in the group with the highest BMI.

**Fig. 3:**
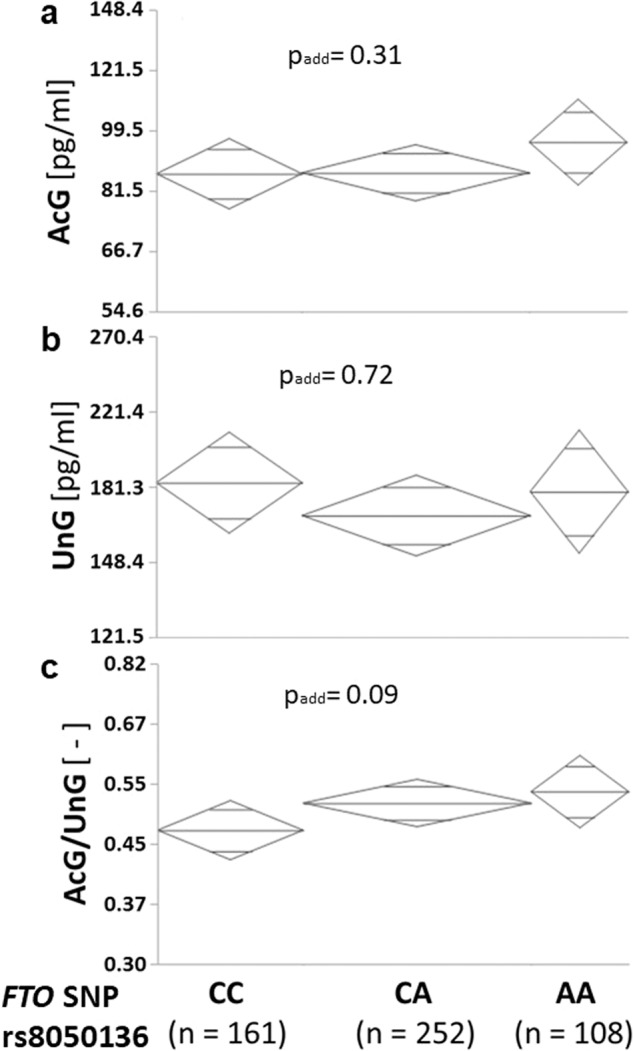
Genetic influence of FTO on ghrelin concentrations. Effect of the *FTO* single nucleotide polymorphism rs8050136 on the fasting levels of acylated ghrelin (AcG; **a**) and of unacylated ghrelin (UnG; **b**) as well as on their ratio (AcG/UnG; **c**) in 521 subjects.

Since single nucleotide polymorphisms (SNP) in the *FTO* gene, which are well known to influence body fat mass and BMI [1, 35, 36], were implicated in ghrelin synthesis and action, we analyzed the effect of the *FTO* genotype rs8050136 on circulating ghrelin concentrations in 521 individuals with available DNA samples using an additive genetic effect model. Univariate analyses (Fig. 3a–c) revealed no genotype-effect on AcG (*p* = 0.31) or UnG (*p* = 0.72) levels, but a trend towards a higher AcG/UnG ratio in minor allele carriers (*p* = 0.089; Fig. 3c). In a multivariate analysis including BMI, minor allele carriers had significantly higher AcG levels (*p* = 0.034). However, the genotype effect on the AcG/UnG ratio was blunted (*p* = 0.31).

## Discussion

This present study was intended for investigation of, first, circulating ghrelin levels (AcG, UnG and their ratio) in Obesity, second, their interdependencies with insulin resistance, third, their kinetic response after a glucose load during an oGTT and, fourth, their association with the *FTO* SNP rs8050136.

Strong negative correlations with BMI were detected for both AcG and UnG levels implicating their distinct declines in adiposity. To our knowledge, our results are the first to reveal a highly significant positive correlation between the AcG/UnG ratio and BMI in a large cohort with appropriately stabilized samples. Both AcG and UnG levels were positively correlated with insulin sensitivity. However, this relation was no longer detectable after adjustment of insulin sensitivity for BMI. In contrast, the correlation with BMI was independent of insulin sensitivity. Thus, multivariate analyses including ISI and BMI favor a superior effect by BMI indirectly inducing an inverse relationship with ISI *via* obesity (leading to insulin resistance and impaired glucose tolerance). If there is a direct relationship between insulin resistance and ghrelin concentrations at all, this may exist for the proportion of AcG/UnG.

Several direct effects of UnG have been reported and support the importance of the AcG/UnG ratio. UnG has been shown to improve muscle oxidative metabolism and performance (Agosti, De Feudis et al. 2020) suggesting GHSR-independent effects of UnG. Furthermore, overexpression of UnG results in improved vascular function (Zanetti, Gortan Cappellari et al. 2019). Inhibition of ghrelin acylation by intraperitoneal administration of a GOAT inhibitor, reducing AcG/UnG ratio, improves glucose tolerance in mice (Barnett, Hwang et al. 2010).

Furthermore, in small interventional studies of caloric restriction in humans UnG increases along with improved glycemia [37]. In line with this observation, an increased AcG/UnG ratio was described for postmenopausal women with insulin-resistance and obesity in comparison to insulin-sensitive controls in a rather small cohort [38] and UnG but not AcG negatively correlated with insulin resistance measured by HOMA index in children with Obesity [39] and adults [30]. Our data supports the observation of a relative excess of AcG or lack of UnG in obesity. However, in our large cohort covering a wide BMI range and precisely derived insulin sensitivity measures in an OGTT, we do not see a major role of the AcG/UnG ratio contributing to insulin resistance in the metabolic syndrome.

Considering the orexigenic effects of ghrelin, the positive correlation between BMI and the AcG/UnG ratio suggests a higher AcG proportion in adiposity. Thus, it appears that the paradoxal reduction of the orexigenic AcG in obesity might be a feedback mechanism to increased dietary caloric intake but not necessarily a pathophysiologic correlate for a reduced orexigenic capability of ghrelin, as this could be determined rather by the AcG/UnG ratio than by the absolute AcG levels. Our observations of decreased absolute AcG and UnG levels but increased AcG/UnG ratio in adiposity might represent an escape mechanism and could explain why obesity mostly persists throughout life although developing obesity is accompanied by reductions in the absolute levels of the orexigenic AcG.

Therefore, inhibition of ghrelin acylation or stimulation of ghrelin deacylation might represent a promising drug target for treatment of obesity, although the reduced absolute AcG levels in obesity don’t suggest this assumption *prima facie*: If these pharmacologic interventions beneficially changed the AcG/UnG ratio towards lower values, they could have therapeutic effects on treatment of obesity despite the reduced absolute AcG levels.

Analysis of ghrelin kinetics during oGTT revealed slight UnG decreases at early and more distinct increases of AcG and the AcG/UnG ratio at later time points. These dynamics were attenuated with incrementing obesity.

As opposed to our results of acute ghrelin regulation during oGTT after a mere glucose load, several smaller studies reported distinct reductions of AcG and UnG levels after ingestion of food consisting of 50% carbohydrates [13], in standard test meal studies [1], after ingestion of a beverage consisting of 80% carbohydrates [19] and during an oGTT [14, 21].

These discrepancies might be explained by the different composition of the respectively applied nourishment (standardized mixed meal [1] *versus* glucose load in our study) as proteins were shown to cause a more profound suppression of ghrelin levels than glucose [19]. Otherwise, an oGTT has been shown to be as effective as a standardized meal by other investigations [20, 40].

However, the studied cohorts were much smaller than the oGTT subcohort of this present project (*N* = 245). And, notably, Foster-Schubert et al. reported biphasical (acylated and total) ghrelin kinetics after ingestion of a primarily (80%) carbohydrate-containing beverage with an initial ghrelin reduction by −65% in the first three hours being followed by subsequent ghrelin increase by 37% (above baseline; after 320 min) in the next three hours [19]. In the present study, in the group of severe obesity (BMI > 40 kg/m³) both, initial suppression as well as later rebound was muted, suggesting a less reactive regulation of ghrelin release in these individuals.

Furthermore, these findings point to a high complexity in the physiological response of AcG levels and UnG levels to an oral glucose load [14].

Delhanty et al. observed that reported AcG levels and thus AcG/UnG ratios in various publications might be too low because the administration of the deacylation inhibitor 4-(2-aminoethyl)benzene sulfonylfluoride hydrochloride (AEBSF) was omitted [14, 41].

The high AcG/UnG ratio in this present study (48% in fasting state) markedly exceeds the acylation ratio of most other publications and indicates adequate preanalytical conditions.

Finally, univariate analyses in our study pointed towards an effect of the *FTO* SNP rs8050136 on the AcG/UnG ratio whereas multivariate analyses rather revealed a relationship with AcG levels. Notably, individuals carrying this *FTO* SNP rs8050136 exhibit an increased fat mass and thus BMI (in consequence of increased food intake, but not of impaired energy expenditure) [42]. Consequently, as our results show a clear positive correlation between BMI and the AcG/UnG ratio, these nonadjusted findings rather seem to be caused by BMI as a confounding variable.

In contrast, the unveiling of a significant association of the *FTO* SNP with increased AcG levels by adjustment for BMI appears plausible, as the *FTO* SNP-associated increased fat mass might lead to the opposing effect of decreased AcG levels. Thus, this increasing effect on AcG-by the *FTO* SNP rs8050136 may be compensatory or rs8050136-associated adiposity may differ from rs8050136-independent adiposity pathobiochemically. As the *FTO* gene product demethylates *N*^6^-methyladenosine, representing a facultative regulatory RNA modification [1], this might be a mechanism to alter the expression of FTO-modulated genes. Furthermore, overexpression of *FTO* in vitro reduced *N*^6^-methyladenosine methylation of ghrelin mRNA increasing both mRNA and peptide levels [1].

This mechanism might contribute to differences in the complex ghrelin actions in the modulation of neurobiochemical processes since homozygous *FTO* SNP carriers exhibit altered neuronal brain activities after presentation of food images along with increased AcG levels as well as reduced postprandial AcG suppression [1].

In conclusion, based on correlation analyses, our results distinctly revealed that both fasting AcG and UnG levels are markedly decreased in obesity with the proportion of active, acylated ghrelin increasing with increasing BMI giving point to a pharmacologic intervention in ghrelin acylation as a treatment of obesity despite decreased absolute AcG levels and /or increase in UnG in patients with high BMI.

In opposition to other reports, we only detected minor effects of AcG and UnG levels in response to an oral glucose intake (instead of profound declines) which were further attenuated with incrementing obesity.

Furthermore, carriers of the common *FTO* SNP rs8050136, exhibit slightly increased AcG levels after adjustment for BMI, and thus might particularly benefit from a therapy leading to increased UnG levels and thus decreased AcG/UnG ratios.

## Data Availability

The datasets analyzed during the current study are not publicly available owing to ethical regulations, but are available from the corresponding author on reasonable request.
